# Stress-Guided Localized Infill Variation for Improving the Mechanical Efficiency of FDM-Printed L-Bracket Structures

**DOI:** 10.3390/polym18192321

**Published:** 2026-09-23

**Authors:** Hussam H. Noor

**Affiliations:** Mechanical Engineering Department, College of Engineering, Taibah University, Madinah 42353, Saudi Arabia; hhnoor@taibahu.edu.sa

**Keywords:** additive manufacturing, polymer, material extrusion, infill, adaptive slicing

## Abstract

Fused deposition modeling (FDM) enables spatial control of internal infill, but uniform infill neglects the nonuniform stress distribution governing failure. This study evaluates a finite element analysis (FEA)-guided, stress-based infill distribution strategy for FDM-printed L-brackets. FEA defined low-, intermediate-, and high-stress regions, implemented as uniform, two-region, and three-region infill configurations. PLA specimens were assessed experimentally in terms of failure load, specimen mass, specific failure load, printing time, and estimated energy consumption. Stress-guided infill distribution increased absolute failure load relative to uniform-infill baselines, but the highest absolute failure load did not necessarily correspond to the most efficient configuration. Mass-normalized analysis demonstrated that moderate two-region configurations achieved the highest specific failure load, outperforming both uniform and three-region configurations. Increasing infill segmentation or excessive density gradients did not yield proportional improvements in mechanical strength. Overall, moderate two-region stress-guided infill distribution provided the most favorable balance between mechanical strength, material usage, and estimated energy demand. The findings demonstrate that FEA-guided infill distribution can improve the efficiency of FDM-printed components rather than simply increasing the overall infill density.

## 1. Introduction

Unlike conventional subtractive and formative manufacturing methods, additive manufacturing (AM) fabricates three-dimensional components through layer-by-layer deposition and consolidation of materials according to a digitally defined geometry of the product [1]. Fused deposition modeling (FDM), one of the most widely adopted AM technologies, is particularly attractive because of its relatively low equipment cost, operational simplicity, and compatibility with a variety of processing materials. During FDM, a thermoplastic filament is melted and extruded through a heated nozzle to accumulate multiple layers, producing a three-dimensional geometrical object [2]. Components can be fabricated as solid, hollow, or partially filled structures, with internal architecture defined by the infill pattern and infill density. Higher infill density generally increases material consumption and build time compared with a hollow component while potentially improving load-bearing capacity and mechanical stiffness [3].

Common infill patterns include honeycomb, grid, and triangular geometries. In general, increasing the infill density enhances the load-bearing capacity, stiffness, and strength of FDM-printed components [4]; however, this improvement is accompanied by increased printing duration, energy consumption, and material usage [5,6,7,8]. Consequently, selecting an appropriate infill density represents a fundamental design trade-off between mechanical performance and manufacturing efficiency. Infill density is often selected empirically or through trial-and-error approaches, frequently resulting in unnecessarily high material usage, long processing duration, and high energy consumption.

In addition to the choice of filament material, multiple FDM processing parameters significantly influence the mechanical performance of additively manufactured components [9,10,11]. Abbas et al. [12] investigated the effect of infill density on the compressive strength of polylactic acid (PLA) specimens and reported a 46% increase when the infill density increased from 20% to 80%, although the printing time increased from 26 to 35 min. Similarly, Arkian [13] reported a positive correlation between PLA infill density and compressive strength. Farazin et al. [6] found that tensile strength increased from 13.8 MPa to 44.1 MPa as infill density increased from 20% to 80%. The average mass of the tested specimens followed a similar trend, increasing from 5.0 g to 10.0 g as the infill density increased. However, higher-density specimens exhibited a more brittle failure, highlighting the balance that must be managed among strength, material usage, and ductility. Gunasekaran et al. [14] studied the effects of varying infill density on the mechanical behavior of FDM-fabricated parts through experiments that characterized hardness, impact strength, tensile strength, and flexural strength. It was found that increasing the infill percentage improved all four characterized responses; however, the percentage improvement differed among the four measured responses. Testing showed that when compared to specimens printed at 25% infill density, specimens printed with 100% infill density had about 36% higher tensile strength, 33% higher hardness, 15% higher impact strength, and 26% higher flexural strength on average. Suteja et al. [15] performed a comprehensive study on the mechanical properties of FDM-printed PLA specimens. The study investigated several infill-related parameters and their effects on the mechanical properties, including infill density, speed of infill deposition, infill pattern, and infill width. The authors determined that out of these four parameters, infill density played a significant role in determining the tensile and yield strength of the test specimens, with increased infill densities yielding better performance. The previous research indicates that spatially varying the internal architecture of FDM components can improve the balance between mechanical performance and material efficiency. Tanveer et al. [5] demonstrated that strategically varying the infill density could increase tensile strength by approximately 3.5% while reducing specimen mass by 5%, highlighting the potential of nonuniform-infill architecture. Their findings also suggested that concentrating higher-density infill near the specimen shell can achieve higher tensile strength. Birosz et al. [16] investigated the effect of varying the infill density using a computer-aided design (CAD) model. They demonstrated the feasibility of stress-informed material redistribution by introducing variable-sized holes according to stress concentration contours; however, the use of non-standardized specimen geometries (small and large holes) limited the comparability and repeatability of their results. Although high-performance engineering materials such as PEEK and ULTEM offer superior strength-to-weight ratios compared to metals, their high processing temperature (PEEK-400 °C, ULTEM-355 °C), requirements (e.g., heated chamber), and substantially greater material cost limited their economic applicability [17,18]. Despite the potential of spatial material optimization, limited work has explored the spatially varying infill densities in standardized, load-bearing FDM components.

Finite element modeling has also been used in FDM studies at both the process and component scales. Process-scale finite element models have been developed to represent extrusion, heat transfer, cooling, and deformation during material deposition. Component-scale modeling can be used to understand loading-induced stress and deformation within printed components [19]. Models of FDM parts at the component scale have been built using homogenized continuum properties, orthotropic material models considering raster angle and build orientation, or explicit geometrical representation of deposited roads, voids, shells, and infill [20,21]. Explicit and G-code reconstructed models are capable of better representing as-printed architectures; however, they demand more geometrical information, meshing effort, and material calibration [21,22]. For design-oriented studies, such simplified solid body models can alternatively be used to qualitatively identify relative load paths and stress concentrations instead of quantitatively predicting the behavior of the printed mesostructure. For instance, Gopsill et al. [23] utilized experimentally evaluated FEA-derived stress fields to inform localized infill generation within geometrically constant structures. This workflow of design-oriented FEA creates a useful bridge between structural demand and spatial distribution of the material, especially if the resulting manufactured designs are physically evaluated.

Previous studies have established that infill density influences the mechanical performance of FDM-fabricated components. Although several investigations have explored spatially varying infill density within a single part, limited work has directly correlated infill distribution with component stress to selectively reinforce critical regions. Generally, infill is varied using two main techniques: by changing the pattern of the infill structure (grid, triangles, lines, and zigzag) and by varying the density of the overall infill within the structure. Accordingly, this study proposes a stress-guided infill methodology in which FEA-derived stress distributions are translated into spatially varied infill density profiles. The objective was to achieve the required structural strength while reducing printing time, material waste, and energy consumption. A load-bearing FDM-fabricated L-bracket was subjected to bending-dominated mechanical loading for experimental evaluation.

## 2. Materials and Methods

### 2.1. Stress-Guided Specimen Design

To evaluate the effect of spatially varying infill densities within a single FDM-printed component, a wall-mounted L-bracket was selected as the test geometry because it represents a common load-bearing application and is widely available in online CAD databases. Its structural configuration is particularly suitable for investigating load-adaptive infill because the applied load generates a bending moment, shear force, and localized stress concentrations. The L-bracket geometry used in this study and its dimensions are presented in Figure 1. For experimental validation, the L-bracket sample was rigidly bolted to a wall, while an eyebolt was attached to the free end of the L-bracket for applying gradually increasing weights until structural failure. The experimental setup is shown in Figure 2.

Under the applied loading condition, the dominant loading mode in the horizontal arm was bending, accompanied by shear and a localized stress concentration near the inner fillet. The loading configuration was chosen such that it would result in a spatially varying stress field from which mechanically critical and nominally low-stress regions can be extracted for creating the stress-guided infill configurations. A three-dimensional static finite element analysis was performed utilizing ANSYS^®^ 2026 R1. The bracket was assumed to be homogeneous, isotropic, and linearly elastic with Young’s modulus of E=2450 MPa and Poisson’s ratio of ν=0.36. The solution was governed by the static equilibrium equation shown in Equation (1) [24]:

(1) Ku=F,

where K represents the global stiffness matrix, **u** represents the nodal displacement vector, and **F** represents the applied load vector. Fixed boundary conditions were applied to the wall interface and mounting pin (Figure 3a), and a downward load of 500 N was applied to the loading pin, which was 40 mm away from the fixed wall. The numerical assembly consisted of the solid bracket and two identical bolts representing the mounting and loading connections. The simulated bolt head had a diameter of 30 mm, matching the diameter of the washer used in the experimental testing. The numerical analysis did not explicitly simulate the nut, washer, or threads. The wall-embedded portion of the mounting bolt was constrained in movement. The back side of the bracket contacting the mounting wall was assigned a compression-only support, representing a rigid wall, allowing for separation while the friction was neglected. A downward force of 500 N was applied to the loading bolt, with its axis located 40 mm from the wall, as illustrated in Figure 3a. To simplify the contact between the three geometries utilized in the analysis, bonded contact conditions were assumed for contacts between the head of the mounting bolt and the back surface of the bracket, between the mounting bolt shank and the hole surface, and between the head of the loading bolt and the top surface of the bracket. Frictionless contact was assigned between the shank of the loading bolt and the hole surface. The contacts idealize the attachments and load transfer to represent the preloads and tightening of the bolts without explicitly modeling them. Therefore, the model utilized in this study is used to provide a general understanding of the general stress distribution in the bracket.

ANSYS 2026 R1 Mechanical mesh was used to discretize the three-dimensional solid model with a program-controlled element formulation and order. The mesh was generated using a global element size of 0.8 mm utilizing ANSYS’s adaptive sizing, using a resolution value of 2. Automatic sizing uses the assigned element size as an initial point and adjusts discretizes edges based on geometry curvature and proximity between features. A mesh sensitivity assessment was performed using nine global element sizes ranging from 3.0 to 0.75 mm. Von Mises stress was evaluated at a fixed probe location near the bracket’s inner fillet. Absolute changes in stress between successive meshes were less than 0.8%, and further refinement from the adopted 0.8 mm mesh to 0.75 mm altered the probe stress by only 0.03%. Hence, the 0.8 mm mesh was selected for the qualitative stress-guided design analysis, which demonstrated limited mesh sensitivity at the monitored location. This generated a mesh with 61,424 elements and 108,054 nodes.

FEA was used solely to determine the relative spatial stress distribution to guide the spatial placement of discrete infill regions. It was not intended to reproduce the exact mechanical response of the final porous structure, nor was it used to calculate the nominal densities assigned to each region.

Figure 3b–d illustrate different perspectives of the resultant stress field contour presented as an isometric view (b), a side view (c), and a cross-section of the side view (d). The 20 MPa limit used in the contour figures improves the visualization of the broader stress distribution and facilitates the stress-guided infill configuration modeling. The cross-sectional view emphasizes the stress experienced by the bracket’s holes. From that, generation of the infill zones took into account the global stress distribution obtained from the FEA resulting from the load application and support regions. These considerations resulted in higher-density regions being applied to the relatively high-stress region around the internal fillet and localized regions around the loading and mounting holes where bearing or fastening stresses occur. The resulting high-stress regions were then translated into simplified discrete infill regions compatible with the slicer modifier-body approach. The geometrical boundaries of these regions were held constant across all stress-guided configurations to ensure consistent zone definition throughout the study. Fixed numerical stress thresholds were not applied. Instead, the terms low, intermediate, and high refer to relative stress levels within the bracket. The region boundaries were based on relative changes in the stress contour, which were then simplified into printable modifier bodies.

Figure 4 shows cross-sectional views of the resulting infill configurations. Note that the color and hatch pattern correspond to assigned density regions rather than stress magnitude as displayed in Figure 3b. The continuous stress field was simplified into manufacturable regions rather than replicating boundary locations of stress contours from the FEA. Three design groups were created. Group one consisted of a baseline uniform-infill configuration, which acted as the reference specimen (Figure 4a). Group two employed two infill density regions, with higher density assigned to the high-stress regions while the rest of the bracket maintained the baseline density (Figure 4b). Group three introduced an intermediate density region between baseline and high-density regions to achieve a smoother spatial transition and assess the impact of finer infill segmentation (Figure 4c).

A range of infill density combinations was explored for each design group to observe the effect of the various stress-guided reinforcement schemes on the mechanical performance of the L-bracket. Table 1 summarizes all configurations tested. Each specimen was labeled according to the number of spatial infill regions and the densities associated with those regions. The nomenclature before the dash corresponds to the number of stress-guided regions, where 1R, 2R, and 3R indicate architectures with one, two, and three regions, respectively. The subsequent numerical values denote infill densities starting with the lowest-stress region and moving toward the highest-stress region. For example, 2R15-30 indicates a two-region design with infill densities of 15% and 30%, respectively. Specimen 3R15-30-50 has three regions with associated densities of 15%, 30%, and 50%. Altogether, ten different specimen configurations were tested. Eight physically identical specimens were produced and tested for each configuration, resulting in a total of 80 specimens.

### 2.2. Sample Fabrication and Experimental Procedure

One parametric CAD model was utilized for all L-bracket specimens such that the external geometries remained constant between experimental groups. The L-bracket geometry was modeled using SolidWorks^®^ 2024. exported in STL format, and then imported into Ultimaker Cura^®^ 5.7.1 for slicing. Note that infill density, as referred to throughout this work, is defined as the nominal print parameter assigned within the slicing software and should not be confused with the physical density of the printed PLA material. Rather, the nominal infill density represents the user-defined fraction of the interior volume to be filled with computer-generated infill lines/triangles. Despite being a standard slicing parameter, implementations and the printed volume fraction of material assigned to the infill can vary among different slicing software due to toolpath generation algorithms, treatment of extrusion width, and compensation overlap with outer shells [25,26]. Thus, all percentages in this work are nominal slicer-assigned infill densities and not experimentally measured local material densities.

Regional infill distributions were created using cylindrical modifier bodies within each stress region as determined by the FEA. Individual nominal infill densities were assigned to each modifier body through the per-model settings available in Ultimaker Cura^®^ 5.7.1. For the fixed zigzag infill pattern used in this study, the assigned infill levels were translated into toolpaths with varied spacing between adjacent infill lines. The resulting G-code was then used to print the specimen using the FDM printer. The complete workflow is shown in Figure 5. As shown in this figure, four translucent cylindrical modifier bodies were used to define the high-stress regions of specimen 2R15-50. These modifier regions were assigned a nominal infill density of 50%, while the remaining internal volume was assigned a nominal infill density of 15%.

Samples were produced under identical processing parameters using an FDM printer (K1, Creality^®^, Shenzhen, China). The only variable was the stress-guided infill density distribution. The filament feedstock utilized was PLA-Basic (PLA-Basic, eSUN^®^, Shenzhen, China), a commercially available PLA filament. Processing parameters can be found in Table 2.

No support structures were used during specimen manufacturing process. Each specimen was printed individually, with its L-shaped side facing the building plate. At a printing speed of 300 mm/s and 100% cooling fan speed, no noticeable defects were observed in the hole overhangs. After visual inspection, mechanical testing was conducted on a custom quasistatic loading apparatus designed to recreate the loading scenario described in Section 2.1. and depicted in Figure 2. Specimens were fixed to a plate using a mounting bolt and a washer. An eyebolt secured to the horizontal arm of the specimen was secured to it using washers and nuts. A tightening torque of 1 N.m was applied to each bolted connection. The external load was applied to the eyebolt using a suspended loading assembly (Figure 6). Load was applied in increments of 50 g with a dwell time of 10 s after each increment to limit dynamic loading effects. Loading continued until complete specimen failure.

The failure load was defined as the cumulative applied load associated with complete specimen failure. Since the loading mass increased in increments of 50 g, the corresponding resolution of the failure load measurement was approximately 0.49 N. Specimens were visually inspected after each load increment for crack initiation or other damage. Eight identical specimens of each configuration were tested.

### 2.3. Evaluation Methodology

To maintain consistent external dimensions, all specimens were modeled with the same external geometry regardless of the infill condition. SolidWorks^®^ 2024 was used to measure the volume of all three defined regions for each of the design groups. Table 3 shows the total specimen volume and the volume of each prescribed region. Fractional volumes of each region were calculated by normalizing to the total specimen volume of 43,288 $mm^{3}$.

Experimentally measured and calculated quantities were used to evaluate the mechanical and manufacturing performance metrics of each specimen. Experimentally measured quantities consisted of the failure load, the mass of each specimen, and the printing time. The experimental failure mass was converted into failure load using the relationship shown in Equation (2) [24]:

(2) F=mg,

where m is the applied mass, and g is the gravitational acceleration taken as $9.81\;m/s^{2}$. The measured quantities were subsequently used to derive the specific failure load (SFL), defined as the failure load normalized by the specimen mass, expressed in Equation (3), where $m_{s}$ denotes the specimen mass, and SFL is reported in N/g [27,28]. SFL provides a normalized measure of load-bearing capability and enables the comparison of structural performance and manufacturing efficiency.

(3) $$SFL=\frac{F}{m_{s}},$$

The experimental results were evaluated using three comparative approaches. First, each stress-guided configuration was compared with the corresponding uniform-infill reference specimen having the same baseline infill density. Second, specimens within the same configuration category were compared to investigate the effect of varying infill densities at a constant number of spatial regions. Finally, two-region and three-region configurations were compared to assess how increasing the number of stress-guided infill zones influences the mechanical performance and manufacturing efficiency of the specimens.

Eight independently produced replicate specimens were printed and tested (n=8) for each of the ten configurations studied, resulting in a total of eighty specimens. The specimens were printed individually using the processing parameters given in Table 2. All specimens were tested using the same quasistatic loading procedure. Results are reported as the mean ± standard deviation. Failure load was treated as the primary mechanical response variable and was assessed separately within the 15% and 30% baseline groups using Welch’s one-way analysis of variance (ANOVA), which does not assume equal group variances. If a significant overall difference was identified, Dunnett’s T3 multiple comparisons test was used to assess the significance between each stress-guided configuration and its respective uniform-infill baseline (1R15 or 1R30). Multiplicity-adjusted p-values less than 0.05 were considered statistically significant. All statistics were performed using GraphPad Prism^®^ 10.6.1.

## 3. Results and Discussion

### 3.1. Failure Load Response

The failure load was first evaluated as the primary mechanical performance metric of the FDM-fabricated L-bracket specimens. It represents the maximum load sustained before structural failure. The results for 15% and 30% baseline infill groups are presented separately in Figure 7, with the dashed horizontal line representing the corresponding uniform-infill reference value. Data are reported as the mean ± standard deviation, with *n* = 8.

Welch’s one-way ANOVA identified significant differences in failure load between configurations for both the 15% baseline specimens [W(5, 19.22) = 2639, p < 0.0001] and the 30% baseline specimens [W(3, 14.91) = 744.1, *p* < 0.0001]. Dunnett’s T3 comparisons revealed that all stress-guided configurations exhibited significantly greater failure loads than the uniform 1R15 reference. Within the 30% baseline specimens, 2R30-50 and 3R30-50-70 had significantly greater failure loads than 1R30, while 2R30-70 had a significantly lower failure load.

For the 15% baseline group (Figure 7a), all stress-guided configurations exhibited higher failure load than the uniform 1R15 reference specimen. Within two-region configurations, the response depended strongly on the assigned density of the high-stress region, indicating that increasing local infill density does not necessarily produce a proportional increase in load-bearing capacity.

A similar, although less pronounced, trend was observed for the 30% baseline group (Figure 7b). The 2R30-50 configuration exhibited the highest failure load, followed by the 3R30-50-70 configuration, whereas 2R30-70 configuration performed below the reference uniform specimen. Compared with the 15% baseline group, the 30% baseline configurations exhibited a smaller relative improvement in the failure load. The results show that the localized infill redistribution can improve the absolute load-bearing capacity of the L-bracket specimens when the combination of densities is appropriately selected [16]. Importantly, the relationship between the assigned infill density and the failure load capacity is not monotonic. Increasing the density of the high-stress region did not consistently produce a corresponding increase in the structural capacity. Configurations involving large-density discontinuities between adjacent regions generally failed to achieve the highest failure loads. This behavior may arise from abrupt stiffness transitions and localized stress redistribution at the interfaces between regions with high and low densities [29]. Therefore, effective reinforcement depends not only on the maximum infill density but also on the spatial compatibility and gradation of neighboring regions. Since enhanced failure load may also be accompanied by increased material usage, the specimen masses and material efficiencies of the configurations are examined subsequently.

The FEA fulfilled its intended purpose by identifying the relative low-, intermediate-, and high-stress regions used to define discrete infill zones. The improved failure loads obtained for the stress-guided specimens experimentally validate the effectiveness of the FEA-guided zoning strategy for material redistribution. It is important to note that the FEA was not intended to reproduce the experimental failure loads quantitatively; rather, it was used to identify relative stress trends and inform the infill zoning strategy. Hence, the experimental validation focused on evaluating the mechanical effectiveness of the resulting infill configurations rather than establishing a direct numerical to experimental correlation.

### 3.2. Specimen Mass and Material Usage

After failure load testing, the mass of each specimen was evaluated to determine the material usage of each infill type. As the outer dimensions of each specimen were maintained constant, the difference in mass correlates to the amount of material that was used to make up the internal structure, or infill. Figure 8 lists the masses as an average ± standard deviation (*n* = 8) and will be used to calculate mass-normalized properties.

As shown in Figure 8, all stress-guided configurations resulted in an increased mass compared to the uniform-infill reference specimen. This resulted from placing higher infill densities in mechanically significant regions of the specimen. The degree to which mass was added varied based on both the assigned infill densities and the volume fraction of each region. Table 3 shows that the high-stress region makes up about 15.5% of the specimen volume, while the intermediate-stress region makes up about 33.7%. As a result, changes to the density of the larger intermediate-stress region can have a notable effect on overall material usage. These findings suggest that improvements in failure load should be considered with the trade-off in material usage [27]. Increasing the load capacity of the specimen does not necessarily improve structural efficiency if much larger increases in specimen mass are needed. As such, the subsequent analysis considers the specific failure load of each configuration achieved by normalizing the failure load by the specimen mass.

### 3.3. Specific Failure Load

The failure load of all investigated infill configurations was normalized with respect to the specimen mass. This specific failure load (SFL), defined as the failure load per unit mass of printed material, can be used as a mass-normalized measure of structural efficiency [27]. This quantity is especially suited to evaluating stress-optimized infill configurations, as it enables differentiating mechanical performance gains due to intelligent material redistribution from gains achieved primarily through added material quantity [28]. Obtained SFL values are shown in Figure 9.

For the 15% baseline group (Figure 9a), 2R15-30 exhibited the highest SFL among all tested configurations, followed by 2R15-50 and 3R15-30-50, respectively. All three configurations exceeded the uniform 1R15 reference, indicating that moderate spatial variation in infill density can improve the load-bearing capacity. In contrast, 2R15-70 exhibited a lower SFL than the reference configuration despite its higher absolute failure load (Figure 7a). Thus, the additional material introduced into the high-stress region did not provide a proportional increase in failure resistance. Among the three-region configurations, configuration 3R15-30-70 demonstrated improved material utilization, whereas configuration 3R15-50-70 produced an SFL comparable to that of the uniform reference.

For the 30% baseline group (Figure 9b), only 2R30-50 exceeded the SFL of the corresponding uniform configuration. Both 2R30-70 and 3R30-50-70 configurations exhibited lower mass-normalized performance, indicating that increasing the local infill density beyond an optimal range did not yield proportional mechanical benefits. This behavior suggests that at a higher baseline infill density, the benefit of localized reinforcement becomes more limited because additional material may contribute primarily to mass rather than load-bearing capacity.

Overall, the SFL results demonstrate that moderate-density transitions provide greater material efficiency than the high-infill configurations. The reduced performance of configurations incorporating 70% infill further suggests that excessive local densification may reduce the effectiveness of the stress-guided infill distribution while increasing the material usage. Therefore, SFL provides a more informative measure of stress-guided infill distribution strategy than failure load alone, as it identifies configurations that improve the mechanical performance without unnecessary material addition. Although three-region configurations achieved high absolute failure loads, the two-region configurations, especially 2R15-30 and 2R30-50, provided the most favorable mass-normalized performance. Subsequent analysis therefore examines the printing time and manufacturing energy to further evaluate the overall manufacturing efficiency of each configuration.

### 3.4. Printing Time and Manufacturing Efficiency

Printing time was evaluated to assess the manufacturing duration associated with each spatially varying infill configuration. In addition to material usage and mechanical performance, the practical effectiveness of a stress-guided infill strategy depends on whether structural improvements can be achieved without a substantial increase in build time. The measured printing durations for all configurations are presented in Figure 10 and compared with those of the corresponding uniform-infill configurations.

All stress-guided configurations exhibited longer printing times than their corresponding uniform-infill references. This behavior is excepted because higher infill densities require additional extrusion paths, increasing the total toolpath length and material-deposition time [30]. For both the 15% and 30% baseline groups, the two-region configurations exhibited a progressive increase in printing time, as the infill density of the high-stress region increased from 30% to 70%. The three-region configurations required substantially longer printing times, with 3R30-50-70 exhibiting the longest printing duration among the investigated configurations. To quantify the performance of each localized infill configuration relative to its corresponding uniform-infill reference, the percentage change in failure load and printing time were defined in this study using Equations (4) and (5), respectively.

(4) $$\Delta F_{i}=\frac{F_{i}-F_{ref}}{F_{ref}}\times 100\%,$$

(5) $$\Delta t_{i}=\frac{t_{i}-t_{ref}}{t_{ref}}\times 100\%,$$

Here, $F_{i}$ is the failure load of the stress-guided configuration. $F_{ref}$ is the failure load of its corresponding reference configuration; $t_{i}$ is the corresponding printing time of the stress-guided configuration; and $t_{ref}$ is the printing time of the corresponding reference configuration. Positive or negative values represent an increase or decrease in failure load or printing time when compared to the corresponding reference value. These normalized quantities allow for direct comparison of the mechanical benefit and printing time penalty associated with each configuration scheme. Table 4 lists the calculated percentage change in failure load and printing time for each configuration relative to its corresponding reference configuration.

From the calculated values shown in Table 4, 2R15-30 offered the best balance of increased failure load to increased print time within the 15% infill baseline. This configuration produced a 52.95% higher failure load with a 12.90% increase in printing time. Similarly, 2R15-50 offered a 44.66% higher failure load but required only a 19.35% increase in printing time. Three-region configurations within the 15% baseline required significantly more printing time while requiring printing time increases greater than their failure load improvements. For the 30% baseline group, 2R30-50 offered the best combination of mechanical improvement and printing duration. The 2R30-70 configuration decreased failure load, whereas 3R30-50-70 provided only an 11.42% improvement in failure load despite a 75.53% increase in printing time. Printing times increased dramatically for configurations where high-infill densities were assigned to large regions of the build. For this reason, improvements in mechanical performance must be considered with respect to the extra fabrication time required. Two-region configurations provided a better balance between mechanical improvement and printing time compared to the three-region configurations. Because the stress-guided infill generation aims to improve the mechanical performance of the component while avoiding unnecessary material use or manufacturing time, a cross-baseline comparison was made between the highest-performing 15% baseline case, 2R15-30, and the 1R30 case. This comparison is used to determine whether optimizing material location can lead to similar or better part efficiency in terms of mechanical performance, print time, and energy consumption when compared to a uniform high-density infill configuration.

Table 5 summarizes the comparison of configurations 1R30 and 2R15-30. Failure load, specimen mass, SFL, printing time, and estimated energy consumption are reported. Energy consumption was calculated assuming a constant printer power of 200 W while neglecting energy consumption required for the initial heating of the build plate and extrusion head. Although the 2R15-30 configuration exhibited a 6.5% lower failure load than 1R30, its mass was 6.1 g, representing a 22.9% reduction. Consequently, the SFL of 2R15-30 was increased by 19.7%, indicating greater load-bearing efficiency per unit mass. This improvement was achieved with a 2.9% increase in printing time and estimated energy consumption. Overall, the cross-baseline comparison demonstrates that stress-guided infill distribution can provide a more efficient structural solution than increasing the uniform-infill density percentage while substantially reducing material consumption using a limited increase in manufacturing time and estimated energy consumption [16].

### 3.5. Comparative Assessment

The stress-guided configurations were compared in terms of failure load, specimen mass, SFL, and printing time. However, the failure load alone was not sufficient to assess structural efficiency because it may increase simply because additional material is deposited. The configurations were therefore evaluated jointly using failure load, specimen mass, SFL, and printing time.

The results show a clear distinction between configurations with the highest absolute strength and those that led to the greatest material efficiency. Within the 15% baseline group, it can be concluded that the configurations with three regions provided the greatest failure load values. Hence, infill distribution across low-, intermediate-, and high-stress regions can enhance absolute load-bearing capacity. These improvements, however, came at the cost of heavier specimens and longer printing times. After normalization by specimen mass, specimen 2R15-30 had the greatest SFL. Thus, a configuration with the greatest absolute failure load may not always be the most structurally efficient configuration [28]. A similar conclusion was reached for the 30% baseline group, where specimen 2R30-50 was found to have the best balance between failure load and the amount of material used.

Simply increasing the assigned infill density of the high-stress region does not necessarily produce a proportional increase in mechanical performance. Configurations with sharp transitions in infill densities, specifically 2R15-70 and 2R30-70, provided only modest improvements compared to the configurations that used moderate reinforcement. This could be due to abrupt changes in effective stiffness and load-carrying capacity at the boundary between neighboring regions with drastically different internal densities. On the other hand, the three-region configurations allowed for an intermediate infill density between the low- and high-density regions, resulting in smoother spatial transitions between regions. Graded structures exhibited higher absolute failure loads but were less material- and time-efficient.

Overall, when considering both mechanical and manufacturability metrics together, the two-region configurations offered the best overall performance. For configurations with a 15% infill baseline, 2R15-30 achieved the highest SFL with relatively small increases to specimen mass and print time. For the 30% baseline group, 2R30-50 gave the best trade-off by increasing both failure load and SFL compared to the uniform 1R30 reference specimen. In general, these results suggest that moderate stress-guided infill gradients performed better than either extreme local infill density or excessive use of infill regions without accounting for the material use and manufacturing time. The optimal design therefore depends on the combined effects of stress distribution, infill density gradients, material allocation, and process efficiency [30].

### 3.6. Comparison with Recent Studies

Localized and graded infill distributions have recently been shown to offer opportunities for improved mechanical performance of FDM-printed structures. Birosz and Andó [16] adjusted the local infill of PLA specimens based on FEA-calculated stress fields. They observed enhanced mechanical resistance of up to 84% when compared with a uniform infill pattern. Alagheband et al. [31] examined several mesh modifiers for localized reinforcement under tensile and fatigue loading, while dos Reis et al. [32] observed improved mechanical strength through geometrically graded infill distributions. While it is difficult to compare numerical results between these works because of differences in specimen geometry, loading conditions, and infill architecture, they all observed improvements to structural performance through the redistribution of infill instead of uniformly increasing the material volume fraction throughout the component.

The present results follow the same general trend but further demonstrate that the correlation between localized infill density and mechanical enhancement is not monotonic. Failure load improvements within the group with a 15% baseline infill ranged from 10.38% for specimen 2R15-70 to 62.99% for 3R15-50-70. Despite utilizing a significantly lower maximum infill density, 2R15-30 exhibited the highest specific failure load of all tested configurations. Similarly, for the group with a baseline infill of 30%, increasing the local infill to 50% (specimen 2R30-50) improved the failure load by 13.00%, but further increasing the local infill density to 70% (specimen 2R30-70) decreased the failure load by 4.39%. Although all three-region configurations exhibited relatively high absolute failure loads, their greater mass and increased printing time decreased their efficiency.

## 4. Conclusions

In this study, stress-guided infill distribution was evaluated to improve the mechanical and manufacturing efficiency of FDM-printed L-brackets. Relative stress regions were determined from a simplified linear-elastic FEA of a homogeneous solid bracket and used to inform the spatial distribution of discrete infill regions. Nominal infill densities for these regions were chosen as variables. Uniform, two-region, and three-region variants were manufactured and tested experimentally for failure load, mass, specific failure load, build time, and estimated energy usage.

Stress-guided infill distribution improved the absolute failure load of the printed L-bracket specimens relative to the corresponding uniform-infill references. Within the 15% baseline group, the three-region configurations achieved the highest absolute failure loads. Within the 30% baseline group, configuration 2R30-50 achieved the highest failure load and outperformed the other configurations in that group. These results suggest that incorporating reinforcement through low-, intermediate-, and high-stress regions increased resistance to failure. However, maximizing failure load did not translate directly to maximizing structural efficiency. Normalizing the failure load to the specimen mass revealed that stress-guided configurations 2R15-30 and 2R30-50 had the greatest efficiency within their respective baseline categories. Together, these findings suggest that the mechanical advantage gained from spatially varying reinforcement is non-monotonic with increasing infill density. Increasing the infill density to the tested 70% level led to more material usage without a meaningful increase in the failure load, potentially due to abrupt transitions between low- and high-infill densities along with inefficient load transfer between adjacent regions. The addition of the intermediate region enhanced the absolute failure load but also increased the specimen mass and print time, resulting in lower overall efficiency.

All stress-guided configurations required longer printing times compared to the uniform reference baseline. The printing time penalty was significantly larger with three-region configurations. A cross-baseline comparison further demonstrated that optimized spatial material distribution can outperform a higher uniform-infill density in mass-normalized structural efficiency while maintaining comparable manufacturing demand. Overall, moderate two-region, stress-guided infill distribution provided the most favorable balance between load-bearing capacity, material usage, and manufacturing demand.

The main quantitative findings of this study are summarized as follows:

- Under a 500 N load, the FEA successfully identified three distinct regions. The volume proportions of the high- and intermediate-stress regions were 15.5% and 33.7%, respectively.
- Relative to their uniform baseline, 2R15-30 and 2R30-50 increased the failure load by 52.9% and 13.0%, respectively.
- Relative to their uniform baseline, 2R15-30 and 2R30-50 increased the printing time by 12.9% and 20.6%, respectively.
- Compared with 1R30, 2R15-30 used 22.9% less material and had a 19.7% higher specific failure load while increasing the printing time and estimated energy consumption by 2.9%.

These results are limited to the studied L-bracket geometry under quasistatic loading and to PLA specimens printed with a zigzag infill pattern. The homogenous, linearly elastic material model used in the FEA neglected the time-dependent viscoelastic behavior of PLA, including creep and stress relaxation. Thus, the FEA results are interpreted as relative stress to guide the infill placement only. Also, the custom loading apparatus did not provide continuous loading rate control or displacement measurements, and the resolution of the failure load was limited to 0.49 N due to the incremental loading procedure. Further studies should evaluate the applicability of the study findings to other geometries, materials, infill patterns, and loading conditions. In addition, energy consumption was estimated by assuming a constant printer power consumption of 200 W and excluding the initial energy required to heat the building platform and extrusion head. Therefore, the reported values should only be interpreted as approximate estimates rather than direct energy measurements. Future work should examine the interfacial fracture mechanisms, stress transfer behavior across infill density transitions, and the robustness of the proposed strategy under additional geometries, loading conditions, and infill transition schemes.

## Figures and Tables

**Figure 1:**
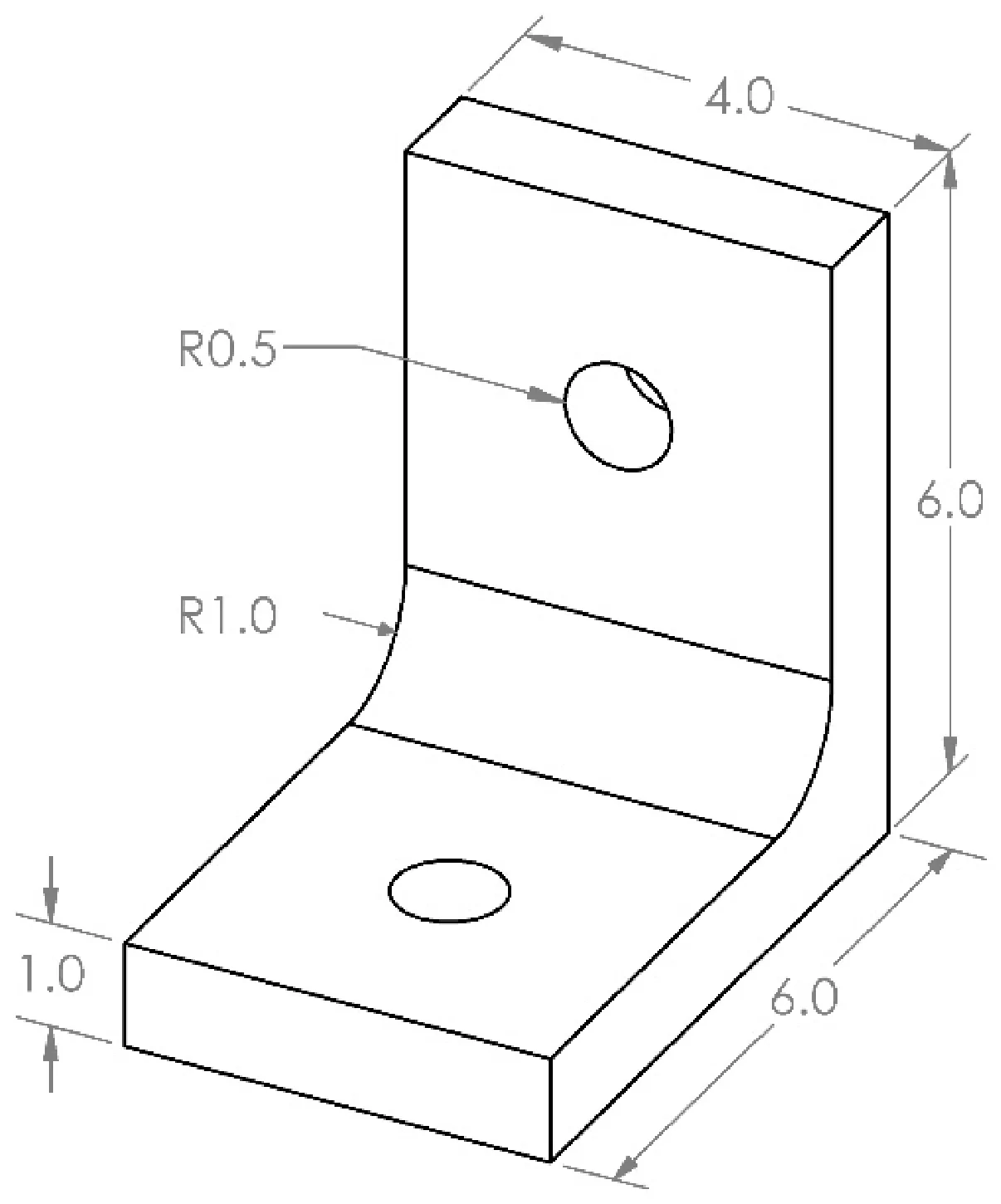
Drawing of the designed part with dimensions in centimeters.

**Figure 2:**
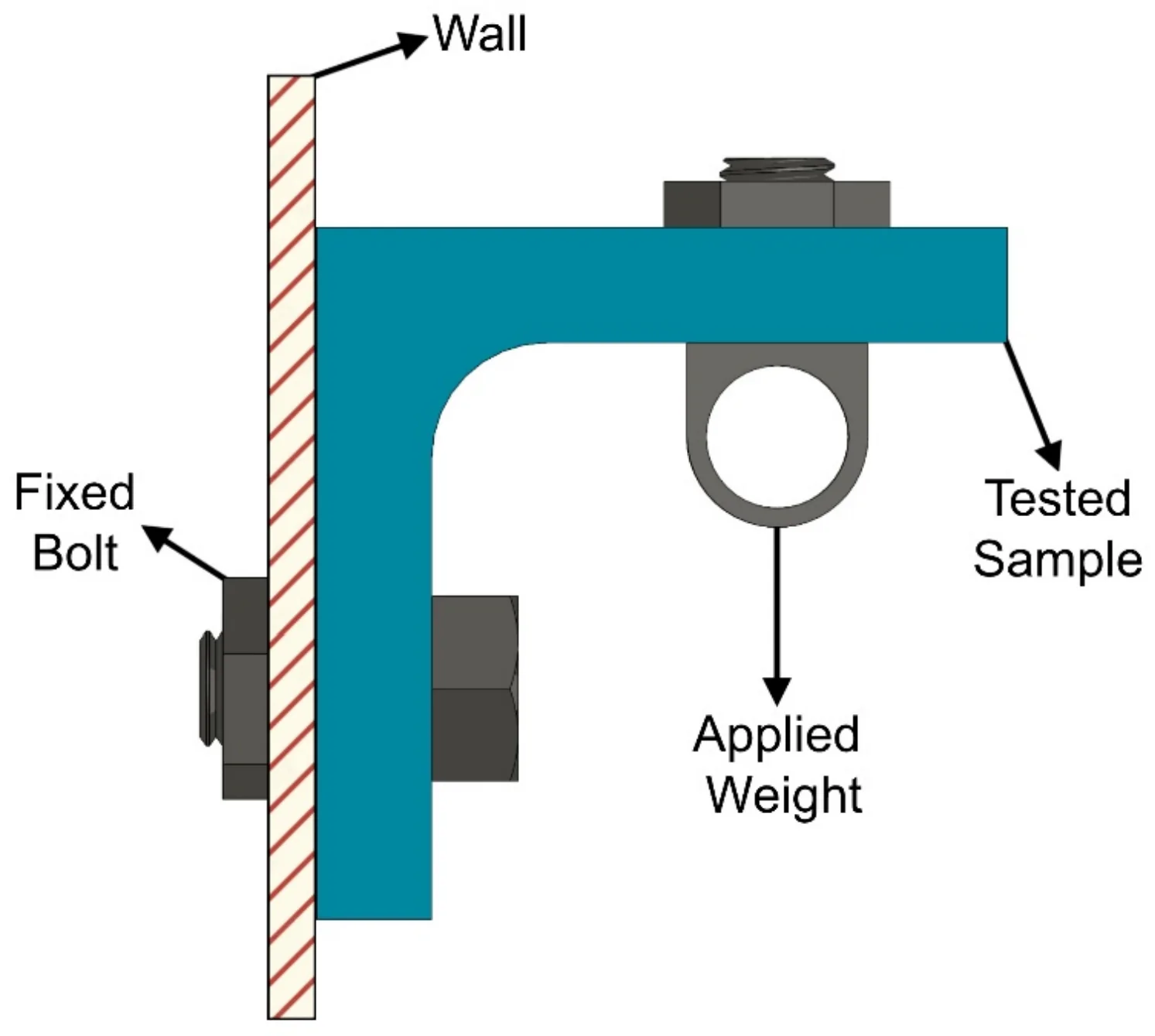
Illustration of the experimental setup with components.

**Figure 3:**
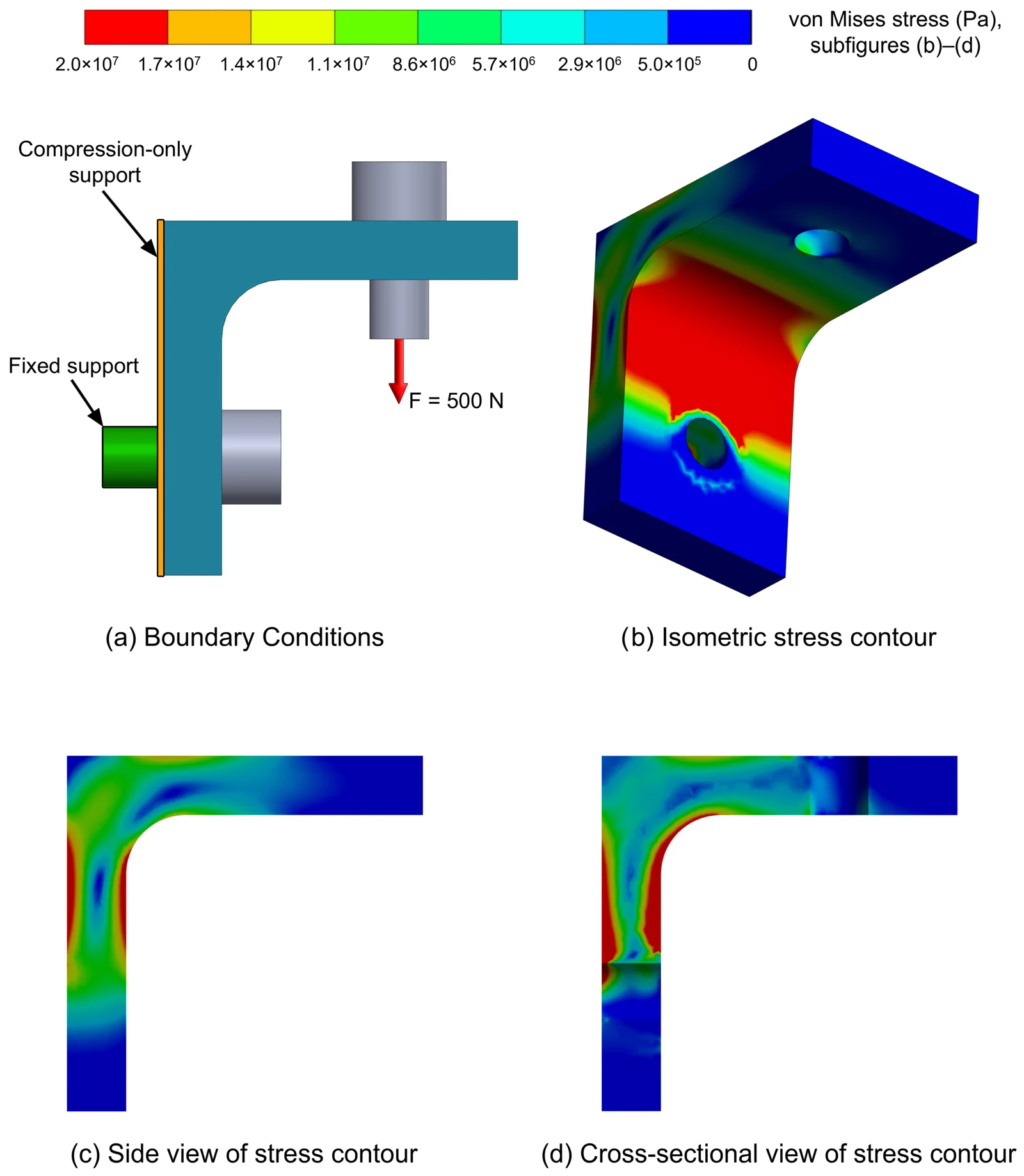
Finite element model and solution of the L-bracket: (**a**) applied force and boundary conditions; (**b**) isometric von Mises stress contour; (**c**) external side view stress contour; and (**d**) sectional stress contour though the bracket mid-width and hole axes. Subfigures (**b**–**d**) share a uniform display scale capped at 20 MPa.

**Figure 4:**
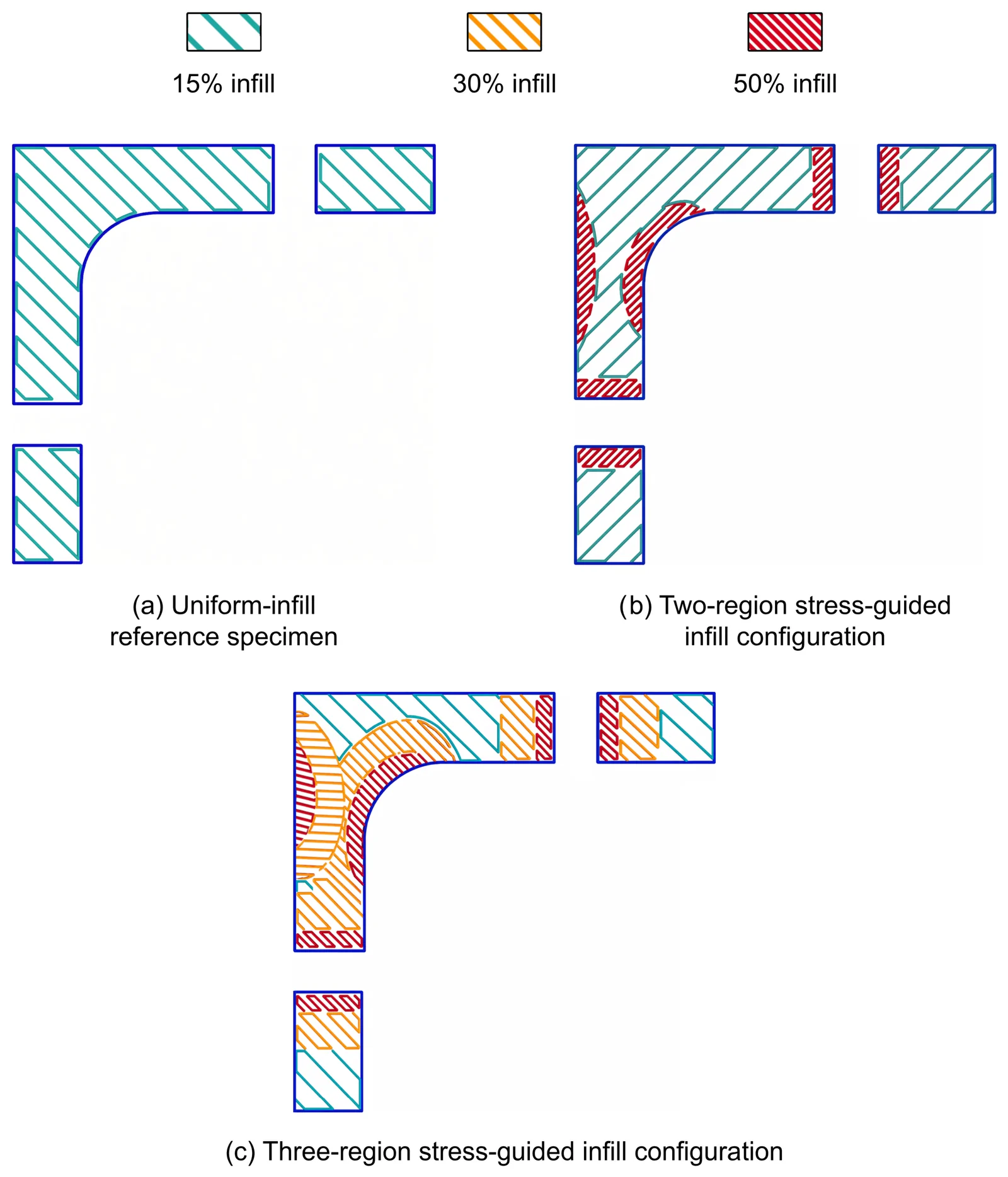
Cross-sectional schematic of uniform, two-region, and three-region infill configurations. The colors and hatch patterns indicate the assigned infill densities rather than stress magnitudes.

**Figure 5:**
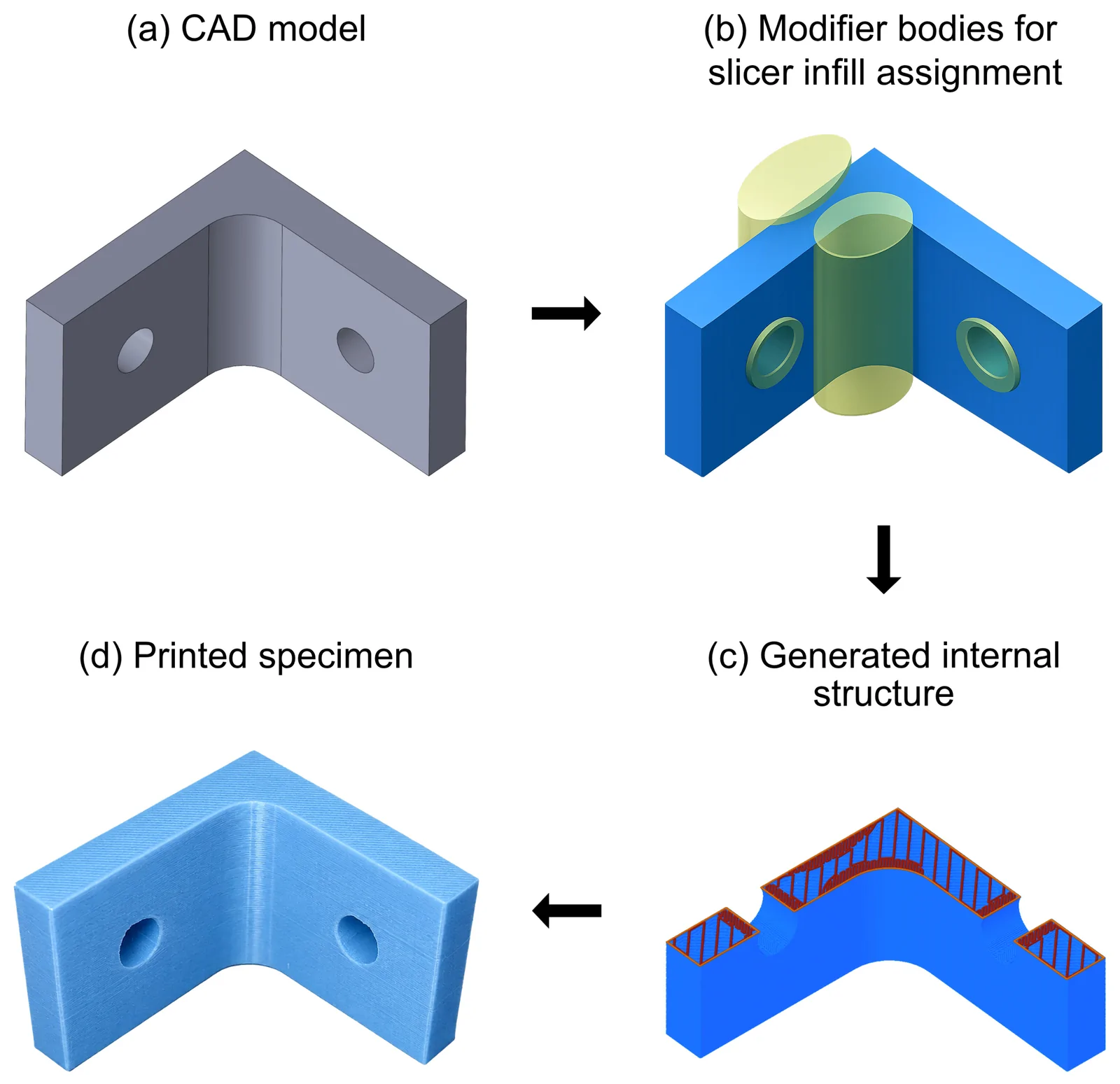
Workflow for generating specimens with region-specific infill densities using slicer modifier bodies. The transparent yellow bodies shown in (**b**) represent the modifier bodies used in the slicing software to alter the infill density of the covered regions.

**Figure 6:**
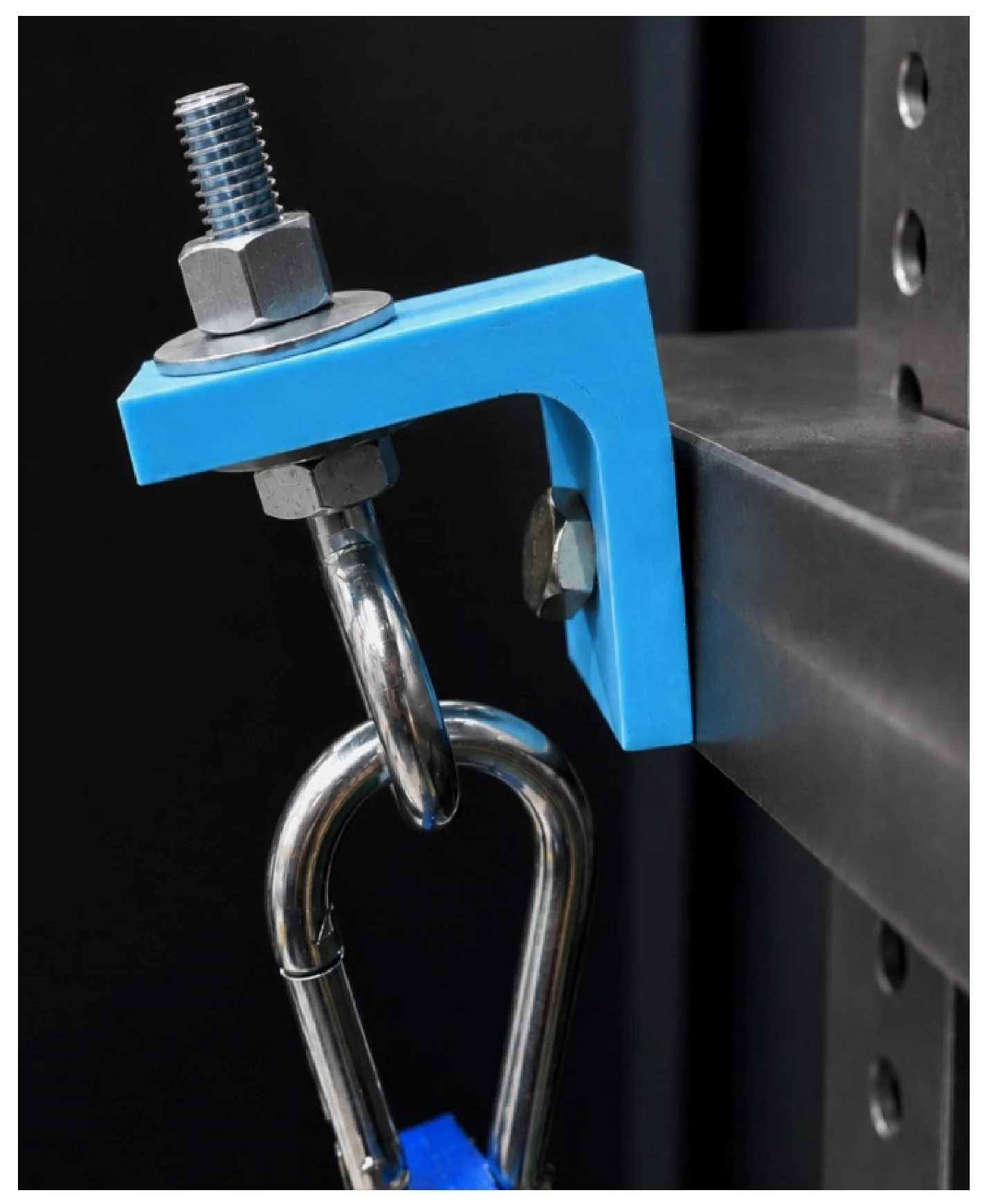
Custom quasistatic loading apparatus used for failure testing.

**Figure 7:**
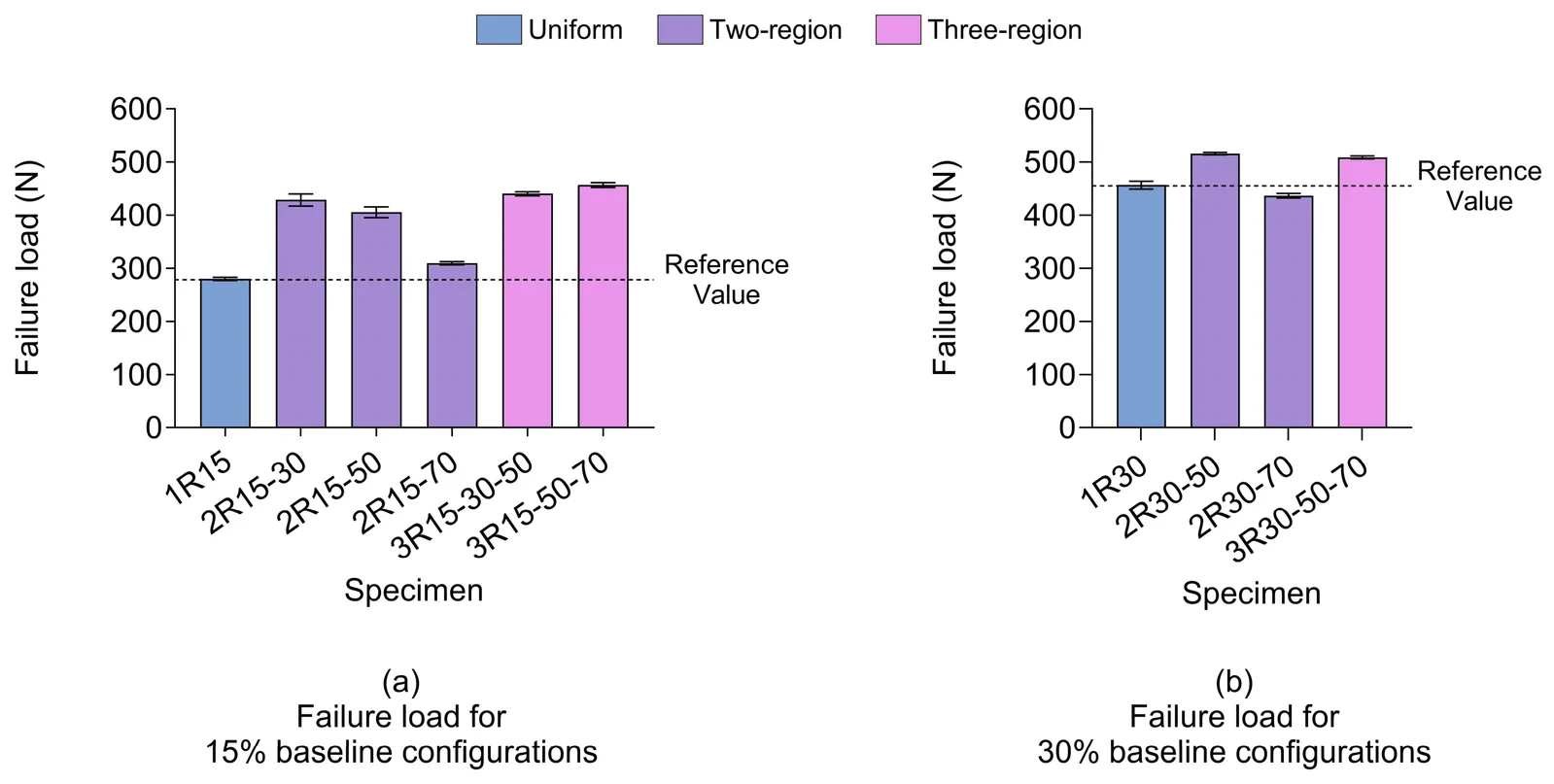
Comparison of the failure loads of stress-guided infill specimens with those of the corresponding uniform-infill reference configurations.

**Figure 8:**
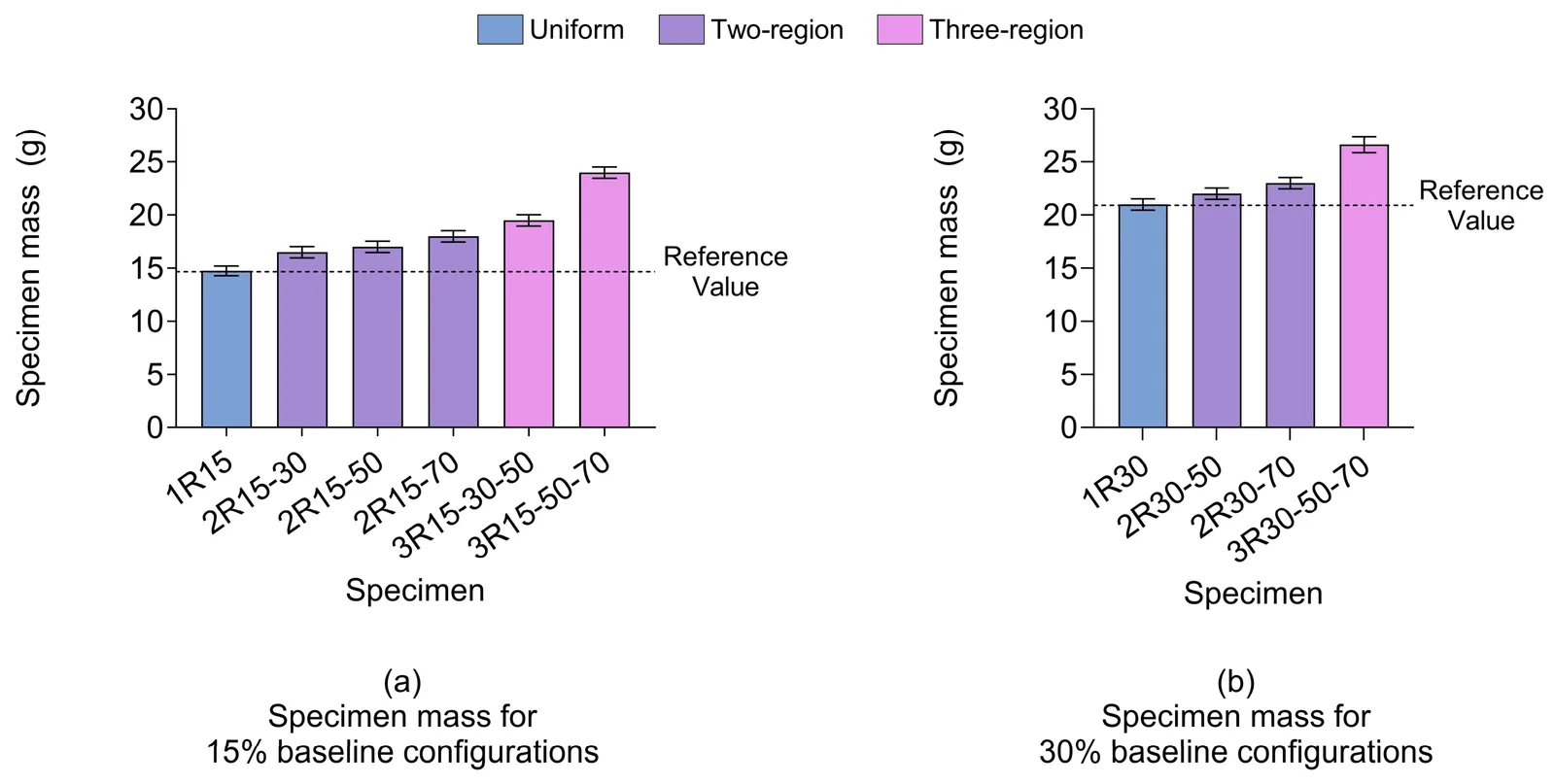
Comparison of the mass of stress-guided infill specimens with those of the corresponding uniform-infill reference configurations.

**Figure 9:**
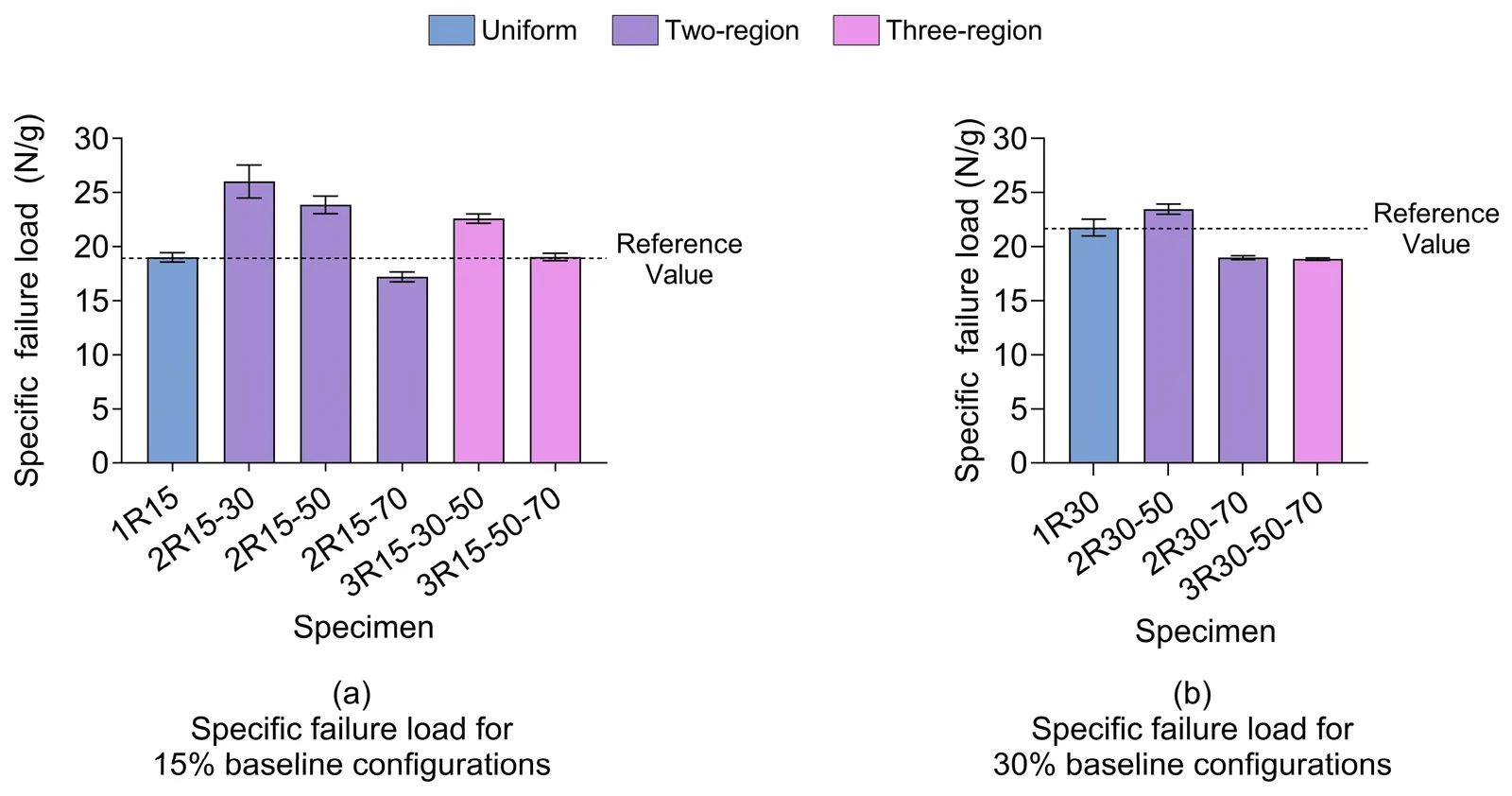
Comparison of the specific failure loads of stress-guided infill specimens with those of the corresponding uniform-infill reference configurations.

**Figure 10:**
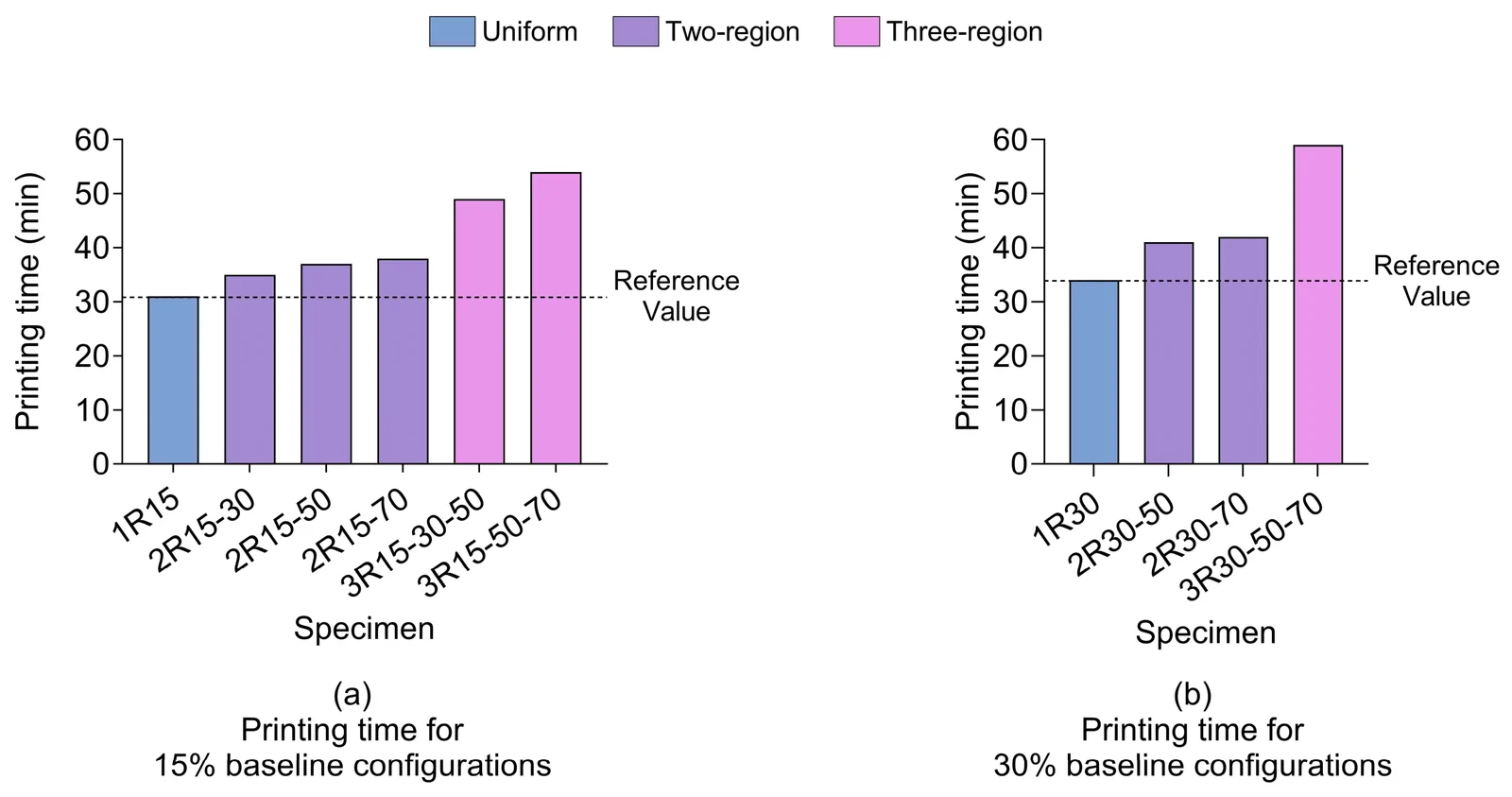
Comparison of the printing time of stress-guided infill specimens with those of the corresponding uniform-infill reference configurations.

**Table 1:** Experimental matrix and specimen nomenclature adopted for specimen configurations.

Specimen ID	Category	Nominal Infill Densities per Stress Region (%)		
		Low	Intermediate	High
1R15	Uniform	15	15	15
1R30	Uniform	30	30	30
2R15-30	Two-region	15	15	30
2R15-50	Two-region	15	15	50
2R15-70	Two-region	15	15	70
2R30-50	Two-region	30	30	50
2R30-70	Two-region	30	30	70
3R15-30-50	Three-region	15	30	50
3R15-50-70	Three-region	15	50	70
3R30-50-70	Three-region	30	50	70

**Table 2:** Fixed printing parameters adopted for specimens.

Parameter	Value
Material	PLA-Basic
Nozzle diameter (mm)	0.4
Filament diameter (mm)	1.75
Nozzle temperature (°C)	220
Heated bed temperature (°C)	60
Number of vertical shells	1
Number of horizontal shells	3
Layer height (mm)	0.2
Printing speed (mm/s)	300
Infill pattern	Zigzag
Nominal infill density (%)	15, 30, 50, and 70
Raster angle (°)	45
Cooling fan (%)	100
Support structure	N/A

**Table 3:** Regional volume distribution of the uniform, two-region, and three-region specimen configurations.

Category	Region	Volume	
		$mm^{3}$	%
Uniform	Whole specimen	43,288	100
Two regions	Low stress	36,592	84.5
	High stress	6696	15.5
Three regions	Low stress	22,005	50.8
	Intermediate stress	14,548	33.7
	High stress	6735	15.5

**Table 4:** Failure load improvement and printing time delay for the stress-guided infill configurations and uniform reference specimens.

Baseline Group	Specimen ID	$\Delta F_{i}$ Failure Load Change (%)	$\Delta t_{i}$ Printing Time Change (%)
15%	2R15-30	52.95	12.90
15%	2R15-50	44.66	19.35
15%	2R15-70	10.38	22.58
15%	3R15-30-50	57.12	58.06
15%	3R15-50-70	62.99	74.19
30%	2R30-50	13.00	20.59
30%	2R30-70	−4.39	23.53
30%	3R30-50-70	11.42	73.53

**Table 5:** Mechanical strength and manufacturing duration of 2R15-30 and 1R30 configurations.

Specimen ID	Failure Load (N)	Mass (g)	SFL (N/g)	Printing Time (min)	Energy Consumption (Wh)
1R30	456.8	20.9	21.8	34.0	113.3
2R15-30	428.9	16.1	26.1	35.0	116.7
Change (%)	−6.5	−22.9	+19.7	+2.9	+2.9

## Data Availability

The datasets supporting the findings of this study are openly available in the Mendeley Data Repository at https://data.mendeley.com/datasets/9dr5zcg9r7/1, accessed on 30 July 2026.

## Abbreviations

The following abbreviations are used in this manuscript:

FDM	fused deposition modeling
FEA	finite element analysis
PLA	polylactic acid
AM	additive manufacturing
SFL	specific failure load
